# New Books

**Published:** 2005-09

**Children’s Health and the Environment: Developing Action Plans**

L. Licari, L. Nemer, G. Tamburlini

Geneva:World Health Organization, 2005. 96 pp. ISBN: 92-890-1374-5, $36

**Computational Genome Analysis: An Introduction**

Richard C. Deonier, Simon Tavaré, Michael S. Waterman

New York:Springer-Verlag, 2005. 540 pp. ISBN: 0-387-98785-1, $79.95

**Deliberative Environmental Politics: Democracy and Ecological Rationality**

Walter F. Baber, Robert V. Bartlett

Cambridge, MA:MIT Press, 2005. 288 pp. ISBN: 0-262-02587-6, $60

**Dynamics of Mercury Pollution on Regional and Global Scales: Atmospheric Processes and Human Exposures Around the World**

Nicola Pirrone, Kathryn R. Mahaffey, eds.

New York:Springer-Verlag, 2005. 748 pp. ISBN: 0-387-24493-X, $129

**Encyclopedia of Biostatistics, 2nd ed.**

Peter Armitage, Theodore Colton, eds.

Hoboken, NJ:John Wiley & Sons, Inc., 2005. 6,100 pp. ISBN: 0-470-84907-X, $2,995

**Environmental Citizenship**

Andrew Dobson, Derek Bell, eds.

Cambridge, MA:MIT Press, 2005. 312 pp. ISBN: 0-262-02590-6, $60

**Generation Extra Large: Rescuing Our Children from the Epidemic of Obesity**

Chris Woolston, Lisa Tartamella, Elaine Herscher

New York:Basic Books, 2005. 255 pp. ISBN: 0-465-08390-0, $25

**Handbook of Urban Health: Populations, Methods, and Practice**

Sandro Galea, David Vlahov, eds.

New York:Springer-Verlag, 2005. 650 pp. ISBN: 0-387-23994, $89.95

**Hazardous Materials Characterization: Evaluation Methods, Procedures, and Considerations**

Donald A. Shafer

Hoboken, NJ:John Wiley & Sons, Inc., 2005. 400 pp. ISBN: 0-471-46251-8, $74.95

**Industrial Transformation: Environmental Policy Innovation in the United States and Europe**

Theo de Bruijn, Vicki Norberg-Bohm, eds.

Cambridge, MA:MIT Press, 2005. 376 pp. ISBN: 0-262-54181-5, $27

**Inventing for the Environment**

Arthur Molella, Joyce Bedi, eds.

Cambridge, MA:MIT Press, 2005. 424 pp. ISBN: 0-262-63328-0, $17.95

**Microarrays for an Integrative Genomics**

Isaac S. Kohane, Alvin Kho, Atul J. Butte

Cambridge, MA:MIT Press, 2005. 326 pp. ISBN: 0-262-61210-0, $25

**Ontologies for Bioinformatics**

Kenneth Baclawski, Tianhua Niu

Cambridge, MA:MIT Press, 2005. 440 pp. ISBN: 0-262-02591-4, $45

**Polluted Promises: Environmental Racism and the Search for Justice in a Southern Town**

Melissa Checker

New York:New York University Press, 2005. 288 pp. ISBN: 0-8147-1658-X, $22

**Power, Justice, and the Environment: A Critical Appraisal of the Environmental Justice Movement**

David Naguib Pellow, Robert J. Brulle, eds.

Cambridge, MA:MIT Press, 2005. 352 pp. ISBN: 0-262-16233-4, $62

**Precautionary Tools for Reshaping Environmental Policy**

Edited by Nancy J. Myers, Carolyn Raffensperger

Cambridge, MA:MIT Press, 2005. 400 pp. ISBN: 0-262-13458-6, $62

**Scarcity and Growth Revisited: Natural Resources and the Environment in the New Millennium**

R. David Simpson, Michael A. Toman, Robert U. Ayres, eds.

Washington, DC:RFF Press, 2005. 320 pp. ISBN: 1-93311-510-6, $70

**Sustainable Development, Energy and the City: A Civilisation of Concepts and Actions**

Voula Mega

New York:Springer-Verlag, 2005. 288 pp. ISBN: 0-387-24354-2, $99

